# Residual Tensile Properties and Explosive Spalling of High-Performance Fiber-Reinforced Cementitious Composites Exposed to Thermal Damage

**DOI:** 10.3390/ma14071608

**Published:** 2021-03-25

**Authors:** Gang-Kyu Park, Gi-Joon Park, Jung-Jun Park, Namkon Lee, Sung-Wook Kim

**Affiliations:** Department of Infrastructure Safety Research, Korea Institute of Civil Engineering and Building Technology, 283 Goyangdae-Ro, Ilsanseo-Gu, Goyang-Si 10223, Korea; gkpark@kict.re.kr (G.-K.P.); joon7767@kict.re.kr (G.-J.P.); jjpark@kict.re.kr (J.-J.P.); nklee@kict.re.kr (N.L.)

**Keywords:** high-performance fiber-reinforced cementitious composites, synthetic fibers, explosive spalling, residual strength, direct tensile strength

## Abstract

This study examined the effect of adding synthetic fibers, that is, polypropylene (PP) and nylon (Ny), on explosive spalling and residual tensile mechanical properties of high-performance fiber-reinforced cementitious composites (HPFRCCs). Three different matrix strengths (100 MPa, 140 MPa, and 180 MPa), four different volume contents of the synthetic fibers (0%, 0.2%, 0.4%, and 0.6%), and three different exposure time (1 h, 2 h, and 3 h) based on the Internatinoal Organization for Standardization (ISO) fire curve were adopted as variables for this experiment. The experimental results revealed that the addition of synthetic fibers improved the resistance to explosive spalling induced by high-temperature, especially when PP and Ny were mixed together. For a higher matrix strength, greater volume content of the synthetic fibers was required to prevent explosive spalling, and higher residual strengths were obtained after the fire tests. An increase in the volume fraction of the synthetic fibers clearly prevented explosive spalling but did not affect the residual tensile strength. In the case of a higher matrix strength, a reduction in the strength ratio was observed with increased exposure time.

## 1. Introduction

To overcome the intrinsic drawbacks of ordinary concrete under tension, many studies [1,2,3] have been conducted to improve the performance of concrete by incorporating several types of fibers, thereby forming fiber-reinforced cementitious composites (FRCCs). Among the FRCCs, a high-performance FRCC (HPFRCC) exhibits a strain-hardening response in tension [4]. This means that even after the initial tensile crack is formulated, the capacity to carry a higher tensile load still remains because of the substantial bridging effect of the fibers between cracks in the cement matrix. Adding fibers leads to enhanced mechanical properties such as matrix strength and fracture energy, especially in the tensile region [5]. Therefore, various HPFRCC production methods, such as engineered cementitious composite (ECC), slurry infiltrated fiber concrete (SIFCON), and reactive powder concrete (RPC), have been developed [2,6,7]. With this improved tensile performance, HPFRC composites have been used under several loading conditions, such as seismic, impact, and blast loading, and under sudden temperature changes [8].

It should be noted that the higher the strength of concrete is, the more vulnerable it is to damage by fire. The higher strength concrete has a denser internal microstructure and low permeability, which leads to the occurrence of spalling [9]. Moreover, in Korea, fire resistance tests should be performed on concrete with a matrix strength greater than 50 MPa, if it is used in residential structures [10]. Therefore, HPFRCCs must be checked for their vulnerability to fire, considering the high performance of composite materials. It has been reported that ECC, unlike other types of HPFRCC, shows better performance in preventing explosive spalling and serious damage under high temperatures, compared with plain mortar. This could be attributed to the fact that ECC is manufactured using polymeric fibers, such as, polypropylene (PP) and polyvinyl alcohol (PVA) fibers, which can alleviate the internal vapor pressure induced by fire [11,12]. The fire resistance of RPC was studied in a previous study [11], in which the residual mechanical properties, pore structures, and explosive spalling were investigated. By performing experiments on cylindrical specimens of ultra-high-performance fiber-reinforced concrete (UHPFRC), a minimum content of PP fibers was suggested to avoid explosive spalling under the given fire scenarios. Zheng et al. [13] tested the fire resistance capacity of RPC with steel fiber content at elevated temperatures. Uniaxial compressive tests were conducted to investigate the influence of the elevated thermal damage on the mechanical properties of RPC, for example, thermal expansion, compressive strength, modulus elasticity, and energy absorption capacity. It was noted that the steel fiber content had an insignificant effect on the mechanical properties of RPC at elevated temperatures. Sahmaran et al. [12] examined the residual characteristics of ECCs with the addition of PVA synthetic fibers after exposure to elevated temperatures for an hour. PVA fibers were found to melt at approximately 200 °C, resulting in additional channels. Therefore, the possibility of explosive spalling was reduced by alleviating the internal vapor pressure. Beglarigale et al. [14] examined the residual flexural strength and change in microstructures in SIFCON exposed to high temperatures. It was reported that the volume fraction of the steel fiber (20%) used in this study could effectively suppress the explosive spalling induced by high temperatures, and the cross-section loss of steel fibers increased with an increase in the thermal damage. Alberti et al. [15] investigated the residual fracture energy and strength of polyolefin fiber reinforced concrete (PFRC) by adopting a three-point bending test after exposure to fire at temperature ranging from 20 °C to 200 °C. Polyolefin macro fibers (PF) are known to be a viable alternative to steel fiber for structural reinforcement in specific applications. It was revealed that the degradation of the mechanical properties of PFRC occurred between 150 °C and 200 °C, and the specimens showed no structural capacity at 200 °C because most of the fibers were found to be melted.

Many studies [11,12,13,14] have been carried out to estimate the residual mechanical properties of HPFRCCs exposed to fire after attaining specific temperatures. However, research [16,17] on the changes in mechanical properties based on the Internatinoal Organization for Standardization (ISO) fire test curve [18] for practical purposes is limited, although it has been reported that the heating rate adversely affects the residual strength of UHPFRC [19]. It is also necessary to investigate the change in the residual properties for various exposure times and matrix strengths. Moreover, the effect of volume content and combination of synthetic fibers on fire resistance should be examined in order to derive the optimal mix design.

In this study, the residual direct tensile properties and explosive spalling of HPFRCCs after fire tests were investigated according to the volume fraction and mixture of synthetic fibers, strength of the cementitious matrix, and fire duration by adopting the ISO standard fire curve. In HPFRCC reinforced with 2% steel fibers, the usage of micro PP and nylon (Ny) was also considered in order to secure fire resistance performance while maintaining the mechanical performance of HPFRCC. The improvement of fire resistance due to the introduction of these synthetic fibers was investigated based on volume contents ranging from 0% to 0.6%. Matrix strengths of 100 MPa, 140 MPa, and 180 MPa and fire durations of 1 h, 2 h, and 3 h were also considered as parameters. After exposing the manufactured specimens to fire, the explosive spalling was examined, and the residual direct tensile properties were analyzed based on the variables.

## 2. Experimental Program

### 2.1. Specimen Preparation

To identify the influence of matrix strength on explosive spalling and residual tensile strength of HPFRCC exposed to fire, three different mix proportions, tabulated in Table 1, were used based on previous studies [20,21]. Various water-binder ratios were adopted to develop different compressive strength levels as the W/B ratio has a considerable effect on the matrix strength [22]. TypeⅠPortland cement, silica fume (SF), ground granulated blast-furnace slag (GGBS), and fly ash (FA) were used as ingredients for the cementitious matrix, and the chemical composition and physical properties of each material are summarized in Table 2. Coarse aggregates were not used because the intention was to improve the homogeneity so as to facilitate a denser internal structure of the cementitious matrix. It has been reported that coarse aggregates interfere with the bonding between the steel fibers and the matrix [23]. Domestic sand numbers 6 and 7 with average sizes of around 294 μm and 174 μm were introduced for the design matrix strengths of 100 MPa and 140 MPa, respectively. Silica sand from, Australia with an average size of around 337 μm was used for the mix design for 180 MPa matrix strength. The mean sizes of TypeⅠPortland cement and SF were 22 μm and 0.31 μm, respectively. Fly ash and GGBS were used for the design matrix strengths of 100 MPa and 140 MPa, respectively. Silica flour with a mean size of 4.2 μm was added as a filler to improve the density of HPFRCC for the 180 MPa matrix strength design, while silica flour with an average size of 14.1 μm was adopted for the 100 MPa and 140 MPa strength designs. Generally, a lower water-to-binder ratio (W/B) is known to result in a higher strength of cement matrix and a relatively lower flowability. To ensure sufficient workability, a polycarboxylate superplasticizer (SP) with a density of 1.01 g/cm^3^ was introduced in this experiment. In addition, a glycol-based shrinkage-reducing admixture (SRA) was used as 0.5% of the weight of cementitious materials to alleviate the potential formation of shrinkage cracks during the production stage.

**Figure 1:**
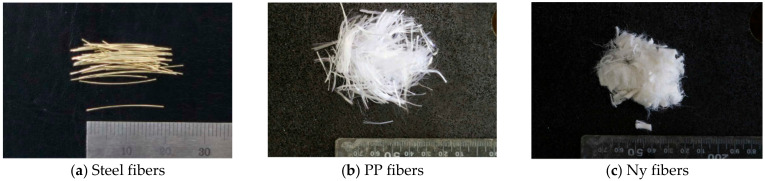
Steel fibers and synthetic fibers are used in high-performance fiber-reinforced cementitious composites (HPFRCC).

Straight steel fibers with 2% volume fraction are included for all the HPFRCC mixtures based on previous studies [24,25] where the volume fraction of steel fibers was limited to 2%, considering the mechanical performance, workability problem, and cost-effectiveness. The steel fibers have an aspect ratio ($l_{f}/d_{f}$) of 97.5, where $d_{f}$ is the diameter and $l_{f}$ is the length of the fiber. In addition, two synthetic fibers, PP and Ny fibers, were used to improve the fire resistance of HPFRCC. The fibers used in this study are presented in Figure 1, and the information and material properties of these fibers are summarized in Table 3. It is widely known that these fibers can alleviate the internal vapor pressure by forming small additional paths during the melting process when the fibers are exposed to high temperatures, improving the fire resistance of HPFRCC. Moreover, according to a previous study [17], a synthetic fiber, which has a higher melting temperature, is more efficient in improving fire resistance. Therefore, in addition to HPFRCC specimens using only PP fibers, samples comprising PP and Ny fibers were fabricated to investigate the improvement in fire resistance because Ny fibers have a thinner diameter and a higher melting temperature than PP fibers. Based on HPFRCC reinforced with 2% steel fibers in order to maintain its mechanical performance, various volume fractions of synthetic fibers (0%, 0.2%, 0.4%, and 0.6%) were considered on an empirical basis because using more than 0.6% volume content of the synthetic fiber resulted in significantly inadequate workability.

**Figure 2:**
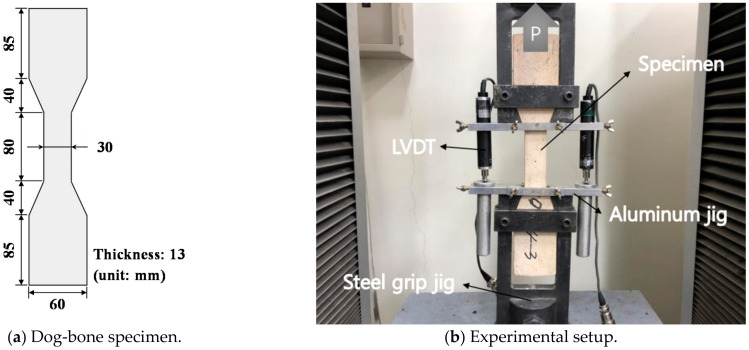
Direct tensile specimen and experimental setup.

After mixing using a Hobart-type mixer, the HPFRCC specimens were cast into a dog-bone shape (see Figure 2). All the specimens were initially cured at room temperature for a day. Thereafter, the demolded specimens were soaked in water at a temperature of 90 °C for 2 days to develop strength. Subsequently, the specimens were again placed at room temperature until the tests were performed. Direct tensile tests were conducted according to the Japan Society of Civil Engineers (JSCE) recommendations [27], and the experimental setup is shown in Figure 2. A monotonic tensile load was applied to a specimen by a universal testing machine (UTM) with a maximum load capacity of 25 t, and the loading rate was set at 0.4 mm/min. A pin-fixed support condition was adopted to minimize the secondary moment, which is recommended by a previous study [28]. Two linear variable differential transformers (LVDTs) connected to an aluminum jig were set up to measure the elongation of the specimens.

The specimens were categorized according to the matrix strength, type and volume content of the synthetic fibers, and fire exposure time. The experimental variables are summarized in Table 4. Five specimens were used for each case, and the average values were tabulated. In the table, the prefix numbers indicate the designed compressive strengths of 100 MPa, 140 MPa, and 180 MPa. The following letters and numbers indicate the type of synthetic fibers and the volume fractions considered in this study, where PP and PN indicate PP fibers and a hybrid of PP and Ny fibers, respectively. The last suffix number represents the heating duration. For instance, 140-PN0.4–2 indicates an HPFRCC direct tensile specimen with 0.4% hybrid fiber content and compressive strength of 140 MPa, subjected to fire for 2 h.

### 2.2. ISO Fire Test

**Figure 3:**
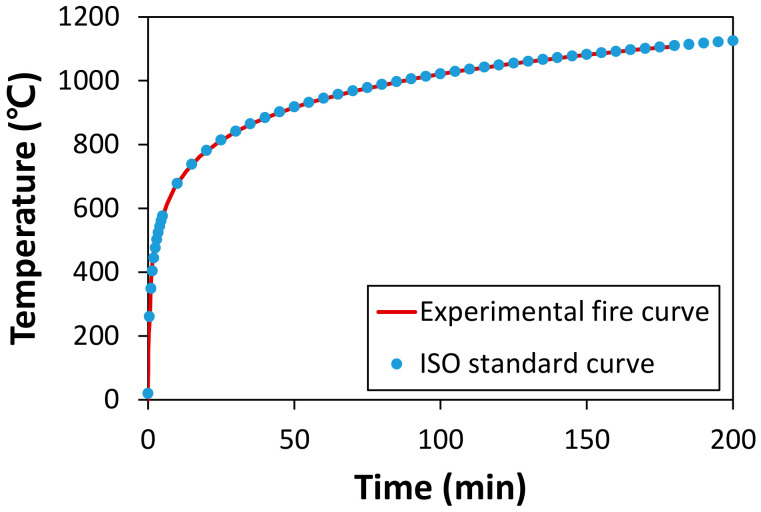
Comparison of actual fire test and ISO curves.

For concrete with a compressive strength greater than 50 MPa, the fire resistance performance should be assessed owing to the possibility of explosive spalling in Korea if such concrete is used for residential purposes [10]. The fire test was carried out according to KS F 2257 [29], which is the same as the ISO 834 standard curve [18]. The temperature curve is expressed as $T=20+345log_{10}\left(8t+1\right)$, where *T* and t mean the actual temperature (°C) in the furnace and the time (min), respectively. The actual temperature in the furnace used in this experiment and the ISO curve is presented in Figure 3. It can be observed that the two curves are almost identical. Fire tests were conducted for the exposure time of 1 h, 2 h, and 3 h. After the tests, the specimens were stored at an ambient temperature for gradual cooling. All the experimental specimens were visually inspected for the occurrence of explosive spalling. The residual strength of the remaining specimens was measured, except for the specimens with explosive spalling.

## 3. Discussion on Experimental Results

### 3.1. Explosive Spalling

The changes in the external appearance of the HPFRCC specimens after the fire tests were examined to investigate the occurrence of explosive spalling based on the matrix strength, volume contents of the synthetic fibers, and fire duration. For HPFRCC specimens without the addition of synthetic fibers, all the specimens were entirely damaged by explosive spalling, irrespective of the matrix strength and fire duration. The vapor pressure in pores induced by high temperatures is not easily alleviated because a high-strength cementitious matrix has an extremely dense internal structure and a lower gas permeability due to a low water-binder ratio and a large number of fine aggregates [30], which results in the explosive spalling. Therefore, HPFRCC specimens, without any additional reinforcement for fire resistance, were seriously damaged by spalling after 1 h of fire exposure, as noted in previous studies [26,31].

**Figure 4:**
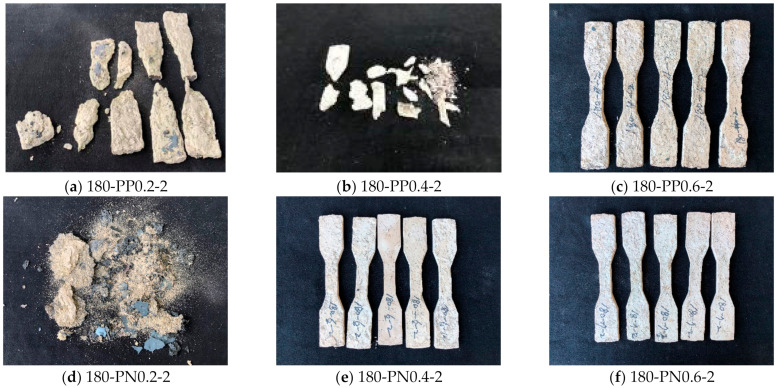
Appearance of the 180 MPa specimens after 2 h of fire exposure.

**Figure A1:**
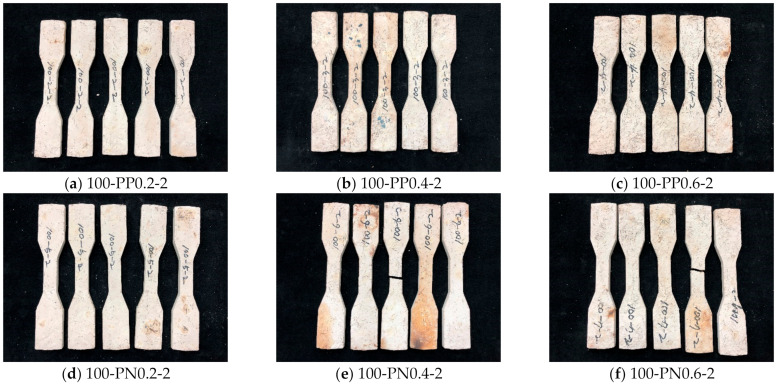
Appearance of the 100 MPa specimens after 2 h of fire exposure.

**Figure A2:**
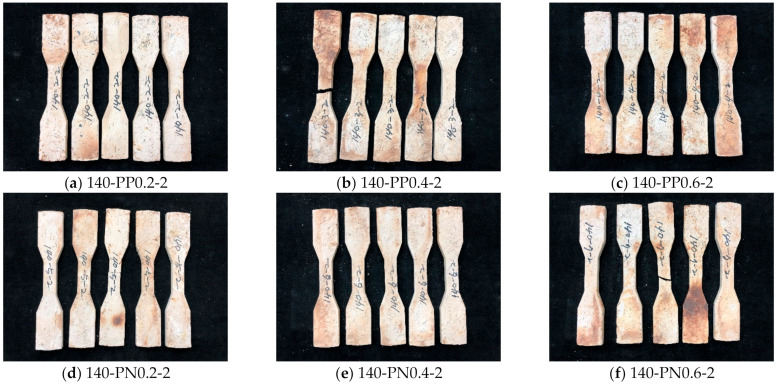
Appearance of the 140 MPa specimens after 2 h of fire exposure.

Furthermore, the resistance to explosive spalling was observed to be improved by the addition of synthetic fibers. As representative experimental results, the images of the specimens after 2 h of fire tests for 180 MPa are presented in Figure 4, and other images with different matrix strengths can be found in Appendix A (see Figure A1 and Figure A2). The color of the specimens gradually turned white as the exposure time increased because of drying and dehydration. Upon the addition of synthetic fibers, overall, the specimens remained intact for the matrix strengths of 100 MPa and 140 MPa, although fractures were observed in a few specimens. From the observation of the specimens with the matrix strength of 180 MPa, it is also obvious that the influence of synthetic fibers on explosive spalling is improved with the increase in the volume fraction. Because vapor pressure could not be released appropriately owing to the low permeability, the specimens with a lower volume content of synthetic fibers were severely damaged for the matrix strength of 180 MPa, causing difficulty in recognizing their original shapes. In particular, severe explosive spalling was observed for the specimens reinforced with 0.4% PP fibers, whereas the specimen reinforced with 0.4% hybrid PN fibers remained intact. This implies that using both PP and Ny fibers is more efficient in terms of explosive spalling than using only PP fibers. A thinner diameter of Ny fibers provides a higher number of fibers per unit volume and then better connectivity between pores by melting when exposed to fire [26,32]. Consequently, it is recommended that at least 0.4% PN fibers and 0.6% PP fibers are required to ensure the explosive spalling of HPFRCC, which is consistent with the previous study [17].

### 3.2. Direct Tensile Strength

**Figure 5:**
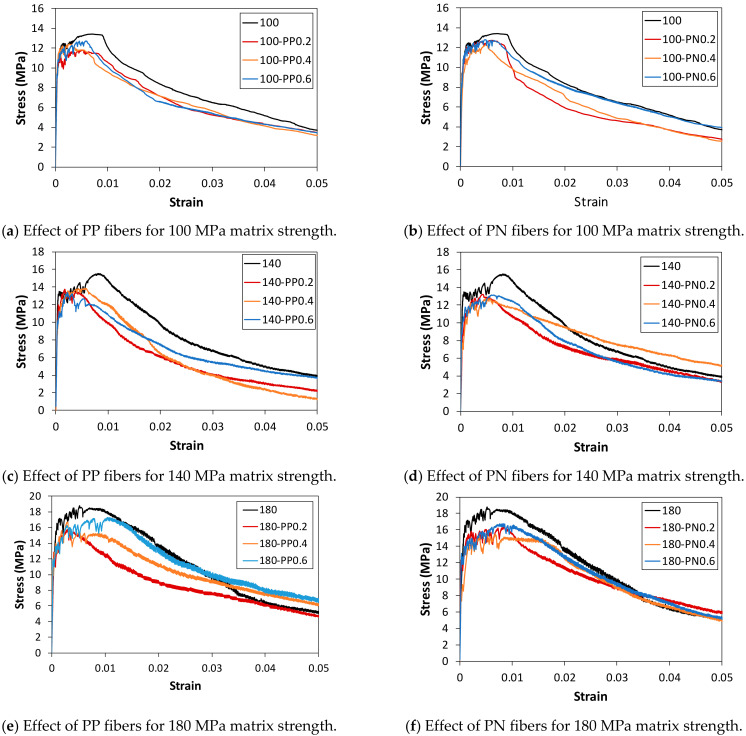
Direct tensile strength before conducting fire tests.

**Figure A3:**
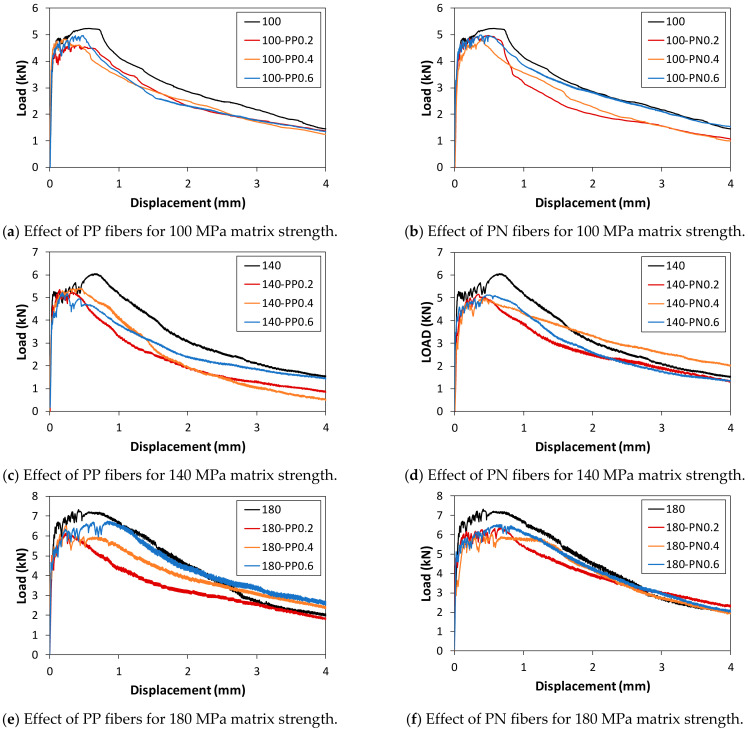
Load–displacement curves before conducting fire tests.

Figure 5 shows the direct tensile stress–strain relations observed from the experiments before the performance of fire tests, and the corresponding load–displacement curves are presented in Appendix A (see Figure A3). The obtained strength values are summarized in Table 5. The tensile strength of the reference specimens without synthetic fibers is included to examine the influence of the synthetic fibers on the direct tensile strength.

By incorporating 2% steel fibers, the results of the reference specimens show a strain-hardening behavior after the formation of initial cracks owing to the bridging effect between the cementitious matrix and steel fibers. Therefore, higher tensile strength is reached. The average tensile strengths of the specimens with the matrix strengths of 100 MPa, 140 MPa, and 180 MPa were measured as 13.41 MPa, 15.53 MPa, and 18.78 MPa, respectively. It can be seen that as the matrix strength increases, the direct tensile strength increases. When the synthetic fibers were additionally used with the HPFRCCs reinforced with 2% steel fibers, the direct tensile strength was slightly reduced. In a previous study [17], it was reported that the flowability of a cementitious mixture decreases with an increase in the addition of synthetic fibers owing to the higher aspect ratio compared with steel fibers. In addition, steel fibers are not easily dispersed because the synthetic fibers interfere with the steel fibers, which reduces the actual number of steel fibers at the cracked surfaces [33,34]. For the same reason, generally, the peak strain is reduced upon the addition of synthetic fibers, compared with that of the specimens reinforced with only steel fibers. In addition to this feature, as shown in Figure 5, it is difficult to differentiate a clear trend induced by the addition of PP and PN fibers and an increase in the volume fraction of the synthetic fibers. Based on the reinforcement mainly using steel fibers in HPFRCC, the additional inclusion of PP and Ny fibers appears to have little effect on the energy absorption capacity in tension due to their relatively weak ability and low volume fraction to prevent crack propagation, as mentioned in a previous study [35].

**Figure 6:**
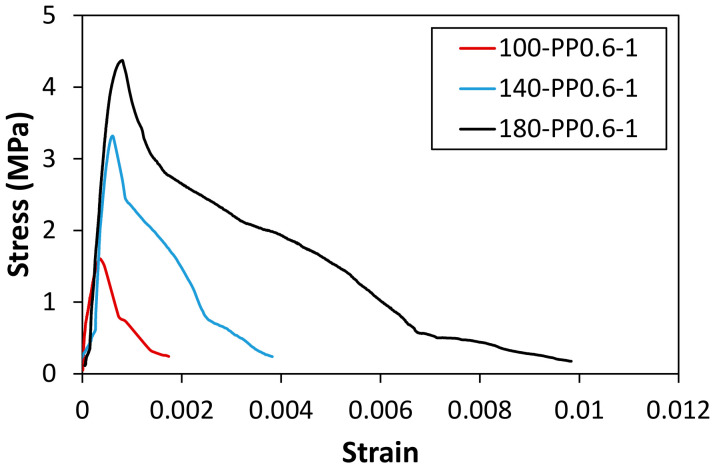
Residual tensile strength for different matrix strengths after 1 h exposure.

**Figure 7:**
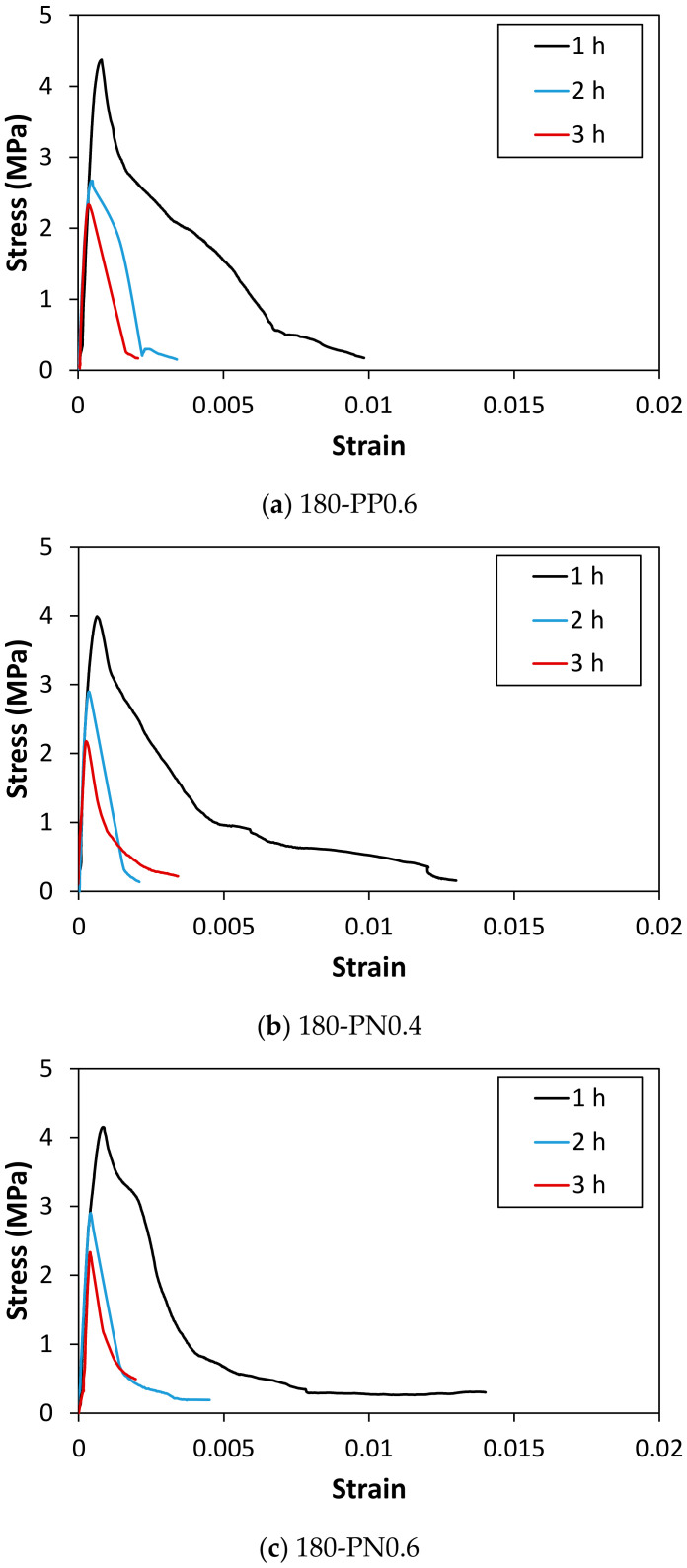
Residual stress–strain curves of specimens with a strength of 180 MPa according to the exposure time.

**Figure A4:**
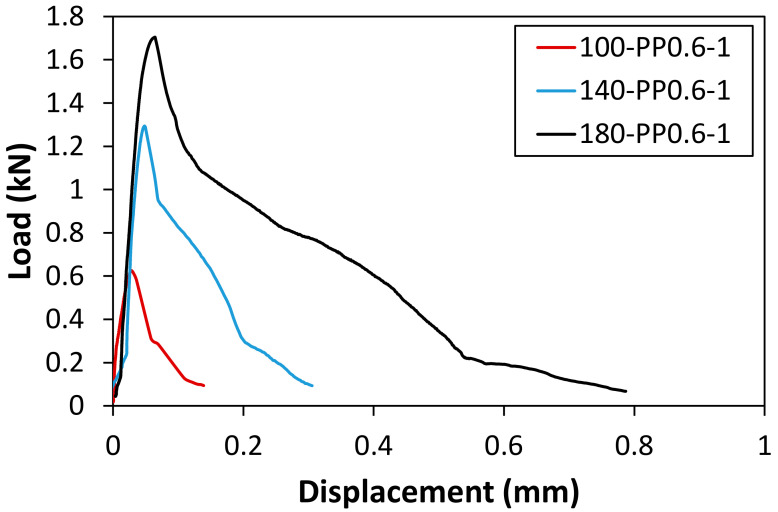
Residual load–displacement curves for different matrix strengths after 1 h exposure.

**Figure A5:**
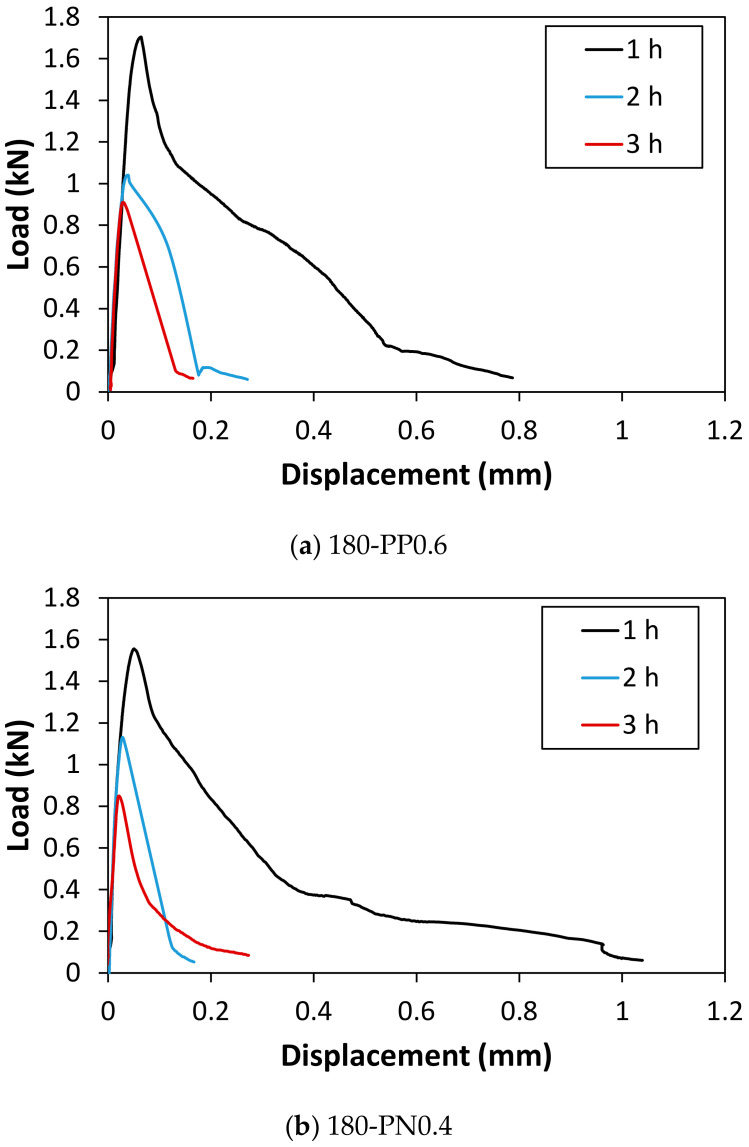

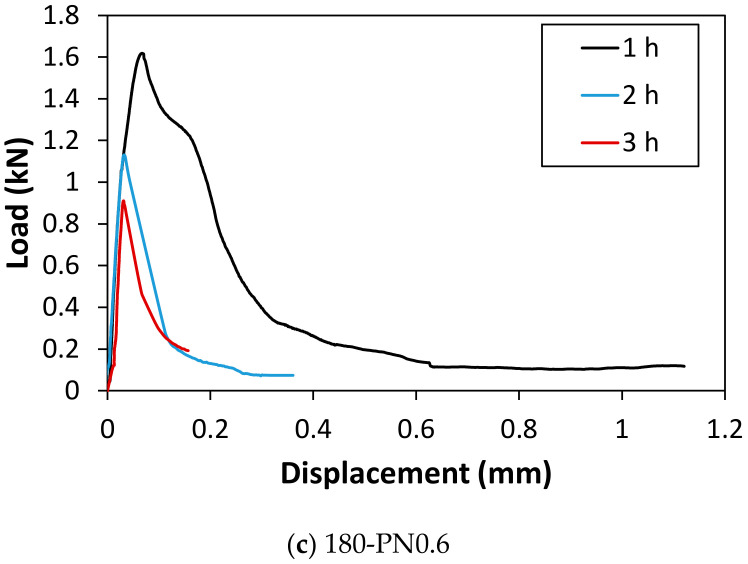
Residual load–displacement curves of specimens with a strength of 180 MPa according to the exposure time.

The representative residual direct tensile stress–strain curves obtained after exposing the specimens to the ISO standard fire are presented in Figure 6 and Figure 7. The corresponding load–displacement curves can be found in Appendix A (see Figure A4 and Figure A5), and the obtained residual strengths are tabulated in Table 5. A few specimens retained their original shape even after the fire tests. However, the direct tensile tests could not be properly performed because there was almost no structural resistance remained in the specimens. Nevertheless, most of the tested specimens endured the fire test regardless of the type and content of the synthetic fibers for the matrix strengths of 100 MPa and 140 MPa, unlike the case for 180 MPa. As shown in Figure 6 and Figure 7, the specimens that endured the fire test did not exhibit any strain-hardening behavior after the fire tests. It was reported in a previous study [36] that steel fibers were oxidized at approximately 800 °C. Under the ISO fire scenario, the actual temperature in the furnace was approximately 900 °C in 1 h. Therefore, it seemed obvious that the synthetic fibers had already melted, and the steel fibers began to oxidize. Moreover, the steel fibers located close to the core of the specimens were more prone to be damaged as the exposure time to fire increased. It is impractical to expect the bridging effect of steel fibers between cracks after the fire tests, and this effect deteriorates as the fire exposure time increases, as shown in Figure 7.

**Figure 8:**
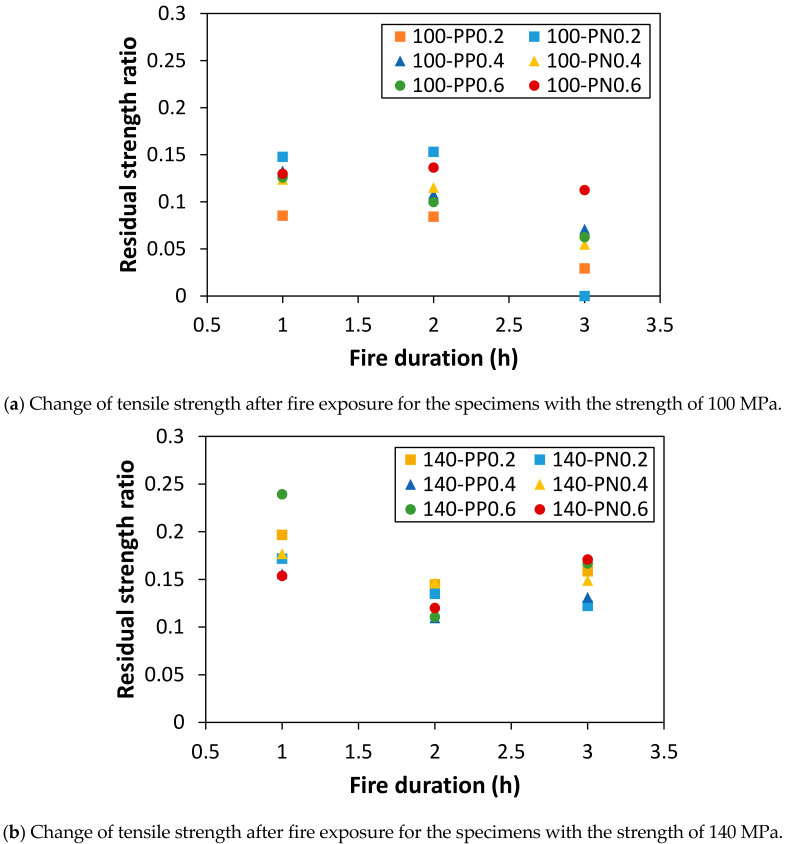

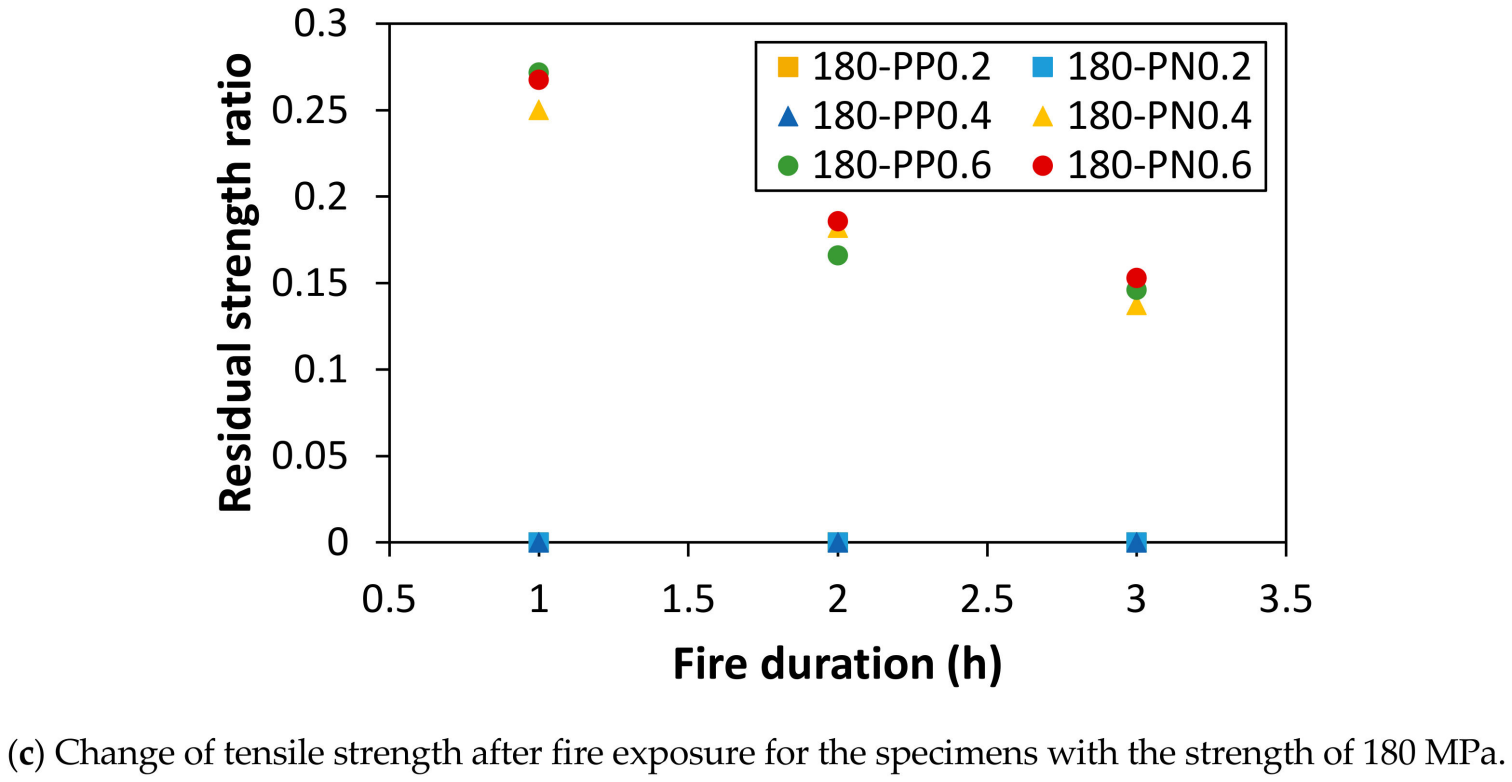
Effect of synthetic-fiber type and fire duration on direct tensile strength.

The ratio of the tensile strength with respect to the results of the specimens without fire damage for each case is presented in Figure 8. A significant reduction in strength can be observed. Further, the residual strength tends to increase as the matrix strength increases (see Figure 6). The reduction in the strength ratio is more noticeable in the case of a higher matrix strength as the fire exposure time increases, compared with the change in the case of a lower matrix strength. This can be attributed to the observation of a lower cumulated porosity with a higher matrix strength when the HPFRCC specimens are exposed to fire for an hour [37]. However, as the exposure time to fire increases, increased numbers of microcracks and voids are generated owing to the internal pressure. The matrix strength noticeably decreases, although a major portion of the internal pressure is alleviated through the channels created by the melting of the synthetic fibers. Furthermore, the decrease in the residual strength ratio with an increase in the exposure time is not noticeable at a lower strength value, owing to the relatively large accumulation of microcracks and porosity after 1 h of fire exposure.

There was no clear trend in the effect of the volume content of the synthetic fibers on the residual tensile strength. This may be because of the thickness of the direct tensile specimens. The specimens were very thin, and the heat from the fire could rapidly reach the core of the sample. Therefore, the temperatures at the core gradually became approximately equal to the surface temperature in a short time, and the synthetic fibers at the core began to melt. Consequently, it is difficult to confirm whether an increase in the volume fraction of the synthetic fibers affects the residual tensile strength if the fibers are reinforced to the extent that explosive spalling does not occur. In addition, it may be considered that the PP and PN fibers do not affect the residual strength because all the fibers melt when exposed to fire. It can be concluded that the synthetic fibers, such as PP and PN fibers, facilitate the HPFRCC specimens to prevent explosive spalling rather than enhance the residual mechanical properties.

## 4. Conclusions

This study investigated the effect of matrix strength, volume contents and combination of the synthetic fibers, and exposure time on explosive spalling and direct tensile properties of HPFRCC based on the ISO standard fire test. In the HPFRCC specimens reinforced with 2% straight steel fibers, PP and Ny fibers were additionally considered to improve fire resistance. After conducting the fire test, explosive spalling was inspected for all the specimens. The residual direct tensile strength was also measured to investigate the strength-reduction ratio according to the variables. The conclusions obtained from this experimental program are as follows:

- The inclusion of the synthetic fibers leads to slightly reduced tensile strength because they interfere with the dispersion of steel fibers. It is also found that the combination and volume fraction of the synthetic fibers in HPFRCC reinforced with 2% steel fibers does not affect the tensile energy absorption capacity;
- The addition of synthetic fibers can effectively improve fire resistance. In particular, the combined use of PP and Ny fibers is more effective in preventing explosive spalling than using only PP fibers. Fabrication of HPFRCCs comprising greater than 0.4% PN fibers and 0.6% PP fibers is recommended in order to avoid explosive spalling and maintain the original shape, considering the high matrix strength;
- The change in the residual strength ratio was prominent for a higher matrix strength with an increase in the exposure time, compared with a lower matrix strength. In addition, a specific trend was not observed in the residual tensile strength according to the volume fraction and combination of the synthetic fibers. Therefore, the main purpose of using the synthetic fibers is to form additional internal passages and alleviate the vapor pressure, thereby lowering the possibility of explosive spalling.

## Figures and Tables

**Table 1:** Mix proportions of the specimens.

ID	$f_{c}\;(\mathrm{MPa})$	W/B	Mix Composition (kg/m^3^)									
			Water	Cement	Silica Fume	Fly Ash	GGBS	Silica Flour	Silica Sand	SP	SRA	Steel Fiber
100	100	0.28	248.4	704.4	70.5	140.9	-	140.9	845.3	11.1	4.6	156.0
140	140	0.25	222.8	739.2	73.9	-	147.8	221.8	813.2	24.3	4.8	156.0
180	180	0.2	163.0	783.6	195.9	-	-	235.1	861.9	47.0	4.9	156.0

**Table 2:** Chemical composition and physical properties of supplementary cementitious materials.

		Cement	Silica Fume	GGBS	Fly Ash
Chemical composition (%)	SiO_2_	21.01	96.00	33.80	65.3
	Al_2_O_3_	6.40	0.25	14.50	16.60
	Fe_2_O_3_	3.12	0.12	0.01	5.58
	CaO	61.33	0.38	42.1	-
	MgO	3.02	0.1	5.80	0.82
	SO_3_	2.15	-	1.89	0.51
	Surface area (cm^2^/g)	3413	200,000	6240	3850
	Density (g/cm^3^)	3.15	2.10	2.91	2.13
	Loss on ignition (%)	1.40	1.50	0.05	3.852

**Table 3:** Information on steel, polypropylene (PP), and nylon (Ny) fibers [17,26].

	Diameter (μm)	Length (mm)	Density (g/cm^3^)	Tensile Strength (MPa)	Elastic Modulus (GPa)	Melting Temperature (°C)
Steel fiber	200	19.5	7.8	2500	200	1538
PP fiber	30	19	0.91	560	1.4–2.2	160.40
Ny fiber	16–28	9	1.1	918	3.9–4.9	220.21

**Table 4:** Summary of experimental specimens.

ID	Volume Fraction (%)/Fiber Dosage (g)			Fire Duration (h)
	Steel Fiber	PP Fiber	NY Fiber	
# (100, 140, or 180)	2.0/32.85			
#-PP0.2-1	2.0/32.85	0.2/0.383	-	1
#-PP0.2-2				2
#-PP0.2-3				3
#-PP0.4-1	2.0/32.85	0.4/0.767	-	1
#-PP0.4-2				2
#-PP0.4-3				3
#-PP0.6-1	2.0/32.85	0.6/1.150	-	1
#-PP0.6-2				2
#-PP0.6-3				3
#-PN0.2-1	2.0/32.85	0.1/0.192	0.1/0.232	1
#-PN0.2-2				2
#-PN0.2-3				3
#-PN0.4-1	2.0/32.85	0.2/0.383	0.2/0.463	1
#-PN0.4-2				2
#-PN0.4-3				3
#-PN0.6-1	2.0/32.85	0.3/0.575	0.3/0.695	1
#-PN0.6-2				2
#-PN0.6-3				3

Note: # indicates the designed compressive strengths of 100 MPa, 140 MPa, or 180 MPa, based on the specimen type.

**Table 5:** Experimental results of the direct tensile strength before and after fire tests.

ID	$f_{t}$ (MPa) (Before Test)	$E_{c}$ (GPa)	Toughness (kJ/m^3^)	$f_{t}$ (MPa) (After Test)		
				1 h	2 h	3 h
100	13.41 (0.076)	34.97 (0.179)	401.9 (0.114)	-	-	-
140	15.53 (0.013)	39.41 (0.306)	442.5 (0.134)	-	-	-
180	18.78 (0.050)	44.61 (0.201)	730.3 (0.221)	-	-	-
100-PP0.2	11.79 (0.113)	28.61 (0.214)	345.2 (0.055)	1.01 (0.305)	0.993 (0.362)	0.35 (0.044)
100-PP0.4	12.40 (0.083)	31.03 (0.174)	341.6 (0.138)	1.65 (0.201)	1.34 (0.081)	0.88 (0.568)
100-PP0.6	12.72 (0.056)	33.36 (0.207)	343.1 (0.041)	1.60 (0.163)	1.27 (0.151)	0.80 (0.421)
100-PN0.2	12.73 (0.142)	32.01 (0.196)	317.5 (0.142)	1.88 (0.170)	1.95 (0.168)	-
100-PN0.4	12.27 (0.123)	31.25 (0.167)	325.6 (0.149)	1.52 (0.192)	1.41 (0.200)	0.67 (0.067)
100-PN0.6	12.79 (0.079)	32.79 (0.341)	384.8 (0.098)	1.66 (0.282)	1.75 (0.072)	1.44 (0.093)
140-PP0.2	13.74 (0.065)	36.76 (0.508)	331.5 (0.166)	2.71 (0.268)	1.99 (0.290)	2.18 (0.184)
140-PP0.4	13.83 (0.127)	37.17 (0.272)	342.8 (0.155)	2.15 (0.263)	1.52 (0.069)	1.81 (0.676)
140-PP0.6	13.37 (0.030)	36.38 (0.296)	340.0 (0.073)	3.20 (0.243)	1.48 (0.191)	2.23 (0.207)
140-PN0.2	13.18 (0.113)	35.79 (0.404)	360.5 (0.114)	2.27 (0.316)	1.78 (0.456)	1.61 (0.543)
140-PN0.4	12.73 (0.127)	38.99 (0.391)	430.9 (0.028)	2.25 (0.452)	1.86 (0.108)	1.90 (0.316)
140-PN0.6	13.14 (0.049)	38.60 (0.296)	375.9 (0.090)	2.02 (0.483)	1.58 (0.368)	2.25 (0.031)
180-PP0.2	15.73 (0.065)	42.48 (0.355)	447.2 (0.221)	-	-	-
180-PP0.4	16.66 (0.092)	40.24 (0.427)	527.3 (0.230)	-	-	-
180-PP0.6	17.29 (0.096)	43.77 (0.430)	584.4 (0.148)	4.37 (0.082)	2.33 (0.186)	2.35 (0.255)
180-PN0.2	16.33 (0.039)	41.35 (0.272)	528.5 (0.116)	-	-	-
180-PN0.4	15.48 (0.036)	42.62 (0.271)	522.1 (0.226)	3.98 (0.068)	2.38 (0.177)	2.19 (0.237)
180-PN0.6	16.72 (0.094)	42.60 (0.421)	549.0 (0.234)	3.87 (0.104)	2.85 (0.171)	2.35 (0.296)

Values in parentheses ( ) mean a Coefficient of variation (CoV).

## Data Availability

Data sharing is not applicable to this article.
